# pH-Sensitive Nanomicelles for Controlled and Efficient Drug Delivery to Human Colorectal Carcinoma LoVo Cells

**DOI:** 10.1371/journal.pone.0100732

**Published:** 2014-06-25

**Authors:** Shi-Ting Feng, Jingguo Li, Yanji Luo, Tinghui Yin, Huasong Cai, Yong Wang, Zhi Dong, Xintao Shuai, Zi-Ping Li

**Affiliations:** 1 Department of Radiology, The First Affiliated Hospital, Sun Yat-Sen University, Guangzhou, China; 2 PCFM Lab of Ministry of Education, School of Chemistry and Chemical Engineering, Sun Yat-Sen University, Guangzhou, China; 3 Department of Medical Ultrasonic, The Third Affiliated Hospital of Sun Yat-Sen University, Guangzhou, China; Texas Tech University Health Sciences Center, United States of America

## Abstract

**Background:**

The triblock copolymers PEG-P(Asp-DIP)-P(Lys-Ca) (PEALCa) of polyethylene glycol (PEG), poly(N-(N’,N’-diisopropylaminoethyl) aspartamide) (P(Asp-DIP)), and poly (lysine-cholic acid) (P(Lys-Ca)) were synthesized as a pH-sensitive drug delivery system. In neutral aqueous environment such as physiological environment, PEALCa can self-assemble into stable vesicles with a size around 50-60 nm, avoid uptake by the reticuloendothelial system (RES), and encase the drug in the core. However, the PEALCa micelles disassemble and release drug rapidly in acidic environment that resembles lysosomal compartments.

**Methodology/Principal Findings:**

The anticancer drug Paclitaxel (PTX) and hydrophilic superparamagnetic iron oxide (SPIO) were encapsulated inside the core of the PEALCa micelles and used for potential cancer therapy. Drug release study revealed that PTX in the micelles was released faster at pH 5.0 than at pH 7.4. Cell culture studies showed that the PTX-SPIO-PEALCa micelle was effectively internalized by human colon carcinoma cell line (LoVo cells), and PTX could be embedded inside lysosomal compartments. Moreover, the human colorectal carcinoma (CRC) LoVo cells delivery effect was verified *in vivo* by magnetic resonance imaging (MRI) and histology analysis. Consequently effective suppression of CRC LoVo cell growth was evaluated.

**Conclusions/Significance:**

These results indicated that the PTX-SPION-loaded pH-sensitive micelles were a promising MRI-visible drug release system for colorectal cancer therapy.

## Introduction

Colorectal carcinoma (CRC) is a malignant disease on the rise. It is the second most common cancer in general and the most common of the gastrointestinal tract cancers [1]. It is currently the second leading cause of death in both female and male patients. Surgical resection is preferred as a curative treatment in early stages of CRC while drug therapy becomes the main preference in advanced or recurrent stages. [2].

In the past decades, a large number of anticancer drugs were identified, although most of them are hydrophobic and poorly soluble in aqeous. Paclitaxel (PTX) is one of the most potent anticancer drugs that can be used in the therapy of several solid tumors. Taxol is one of the formulations of PTX with stratified aqueous solubility and lower toxicity [3], [4]. Nevertheless, the clinical use of PTX is limited by high toxicity and low bioavailability [5]. PTX has been found to have side effects including neurotoxicity, hypersensitivity reactions, cardiotoxicity, and nephrotoxicity [6]. Moreover, for PTX, it has been found that the concentration of drug in tumor is rather low. Its use has remained limited due to this unsatisfactory therapeutic efficacy [7]. In comparison, nanoparticles, such as cationic polymers and cationic peptides loaded with anti-tumor drugs, have potential to overcome these shortcomings. These various carriers had been widely studied for targeted tumor therapies. In fact, the release of a drug in plain carriers usually lasts for days or even weeks. Even if effective delivery to tumor tissue and cells is achieved, it is still difficult to achieve an ideal therapeutic effect and reduce the probability of drug resistance [8]. As a result, the amount of released drug is a key for chemotherapeutic agents to efficiently kill the cancer cells and studies involving rapid and adequateh intracellular drug release from nanocarriers are of particular importance at present.

In recent years, nanocarriers with a triggered release mechanism have been developed, such as micelles which could release drugs in response to specific stimuli such as temperature, pH, ultrasound and redox potential, etc [9]–[12]. Among these carriers, pH-sensitive polymers appears to be the most attractive candidate because the smart delivery systems are stable and can self-assemble in physiological environment (blood, pH = 7.4) but dessembles and releases drug in acidic environments (lysosomal, pH = 5.0), resulting in significantly enhanced anti-tumor efficacy, minimal drug resistance and side effects [13], [14].

Meanwhile, magnetic resonance imaging (MRI) techniques have great advantages in monitoring targeting events and therapeutic outcomes noninvasively. MRI-visible nanoparticulate systems have been used for anticancer drug delivery and imaging of cancer [15]–[17]. Superparamagnetic iron oxide (SPIO), known as a highly efficient T2 contrast agent for MRI, is an ideal small molecular probe. The use of nanocarrier loaded with SPIO can make the vector MRI-visible [16], [17]. However, reports on anticancer outcome of stimuli-sensitive SPIO-PTX-loaded polymeric vesicles with a combined feature of intracellular drug release and imaging function are very rare.

In this study, we developed a nano-micelle for anti-tumor drug delivery and intracellular drug release triggered by pH. The polymeric micelle was designed based on polyethylene glycol (PEG), poly (N-(N’, N’-diisopropylaminoethyl) aspartamide) (P(Asp-DIP)) and poly (lysine-cholic acid) (P(Lys-Ca)). The copolymer PEG-P(Asp-DIP)-P(Lys-Ca) (PEALCa) can self-assemble into nano-scale vesicles encapsulating PTX and SPIO in aqueous solution at neutral pH, and disassembles in acidic lysosomal compartments resulting in rapid drug release. In order to demonstrate the targeting and therapeutic potential of the vesicle, cell culture experiments of the PTX-SPIO-loaded vesicles against human CRC LoVo cells were conducted. Besides, as an MRI-visible drug delivery system, the intracellular drug release of the vesicles *in vivo* was studied by MRI to validate the efficiency. Our study attempts to explore the transferring efficiency of PTX-PEALCa into tumoral cells and and compare the degree of human CRC LoVo cell growth suppression between PTX-PEALCa with conventional anti-cancer drugs.

## Materials and Methods

### Materials

**Figure 1:**
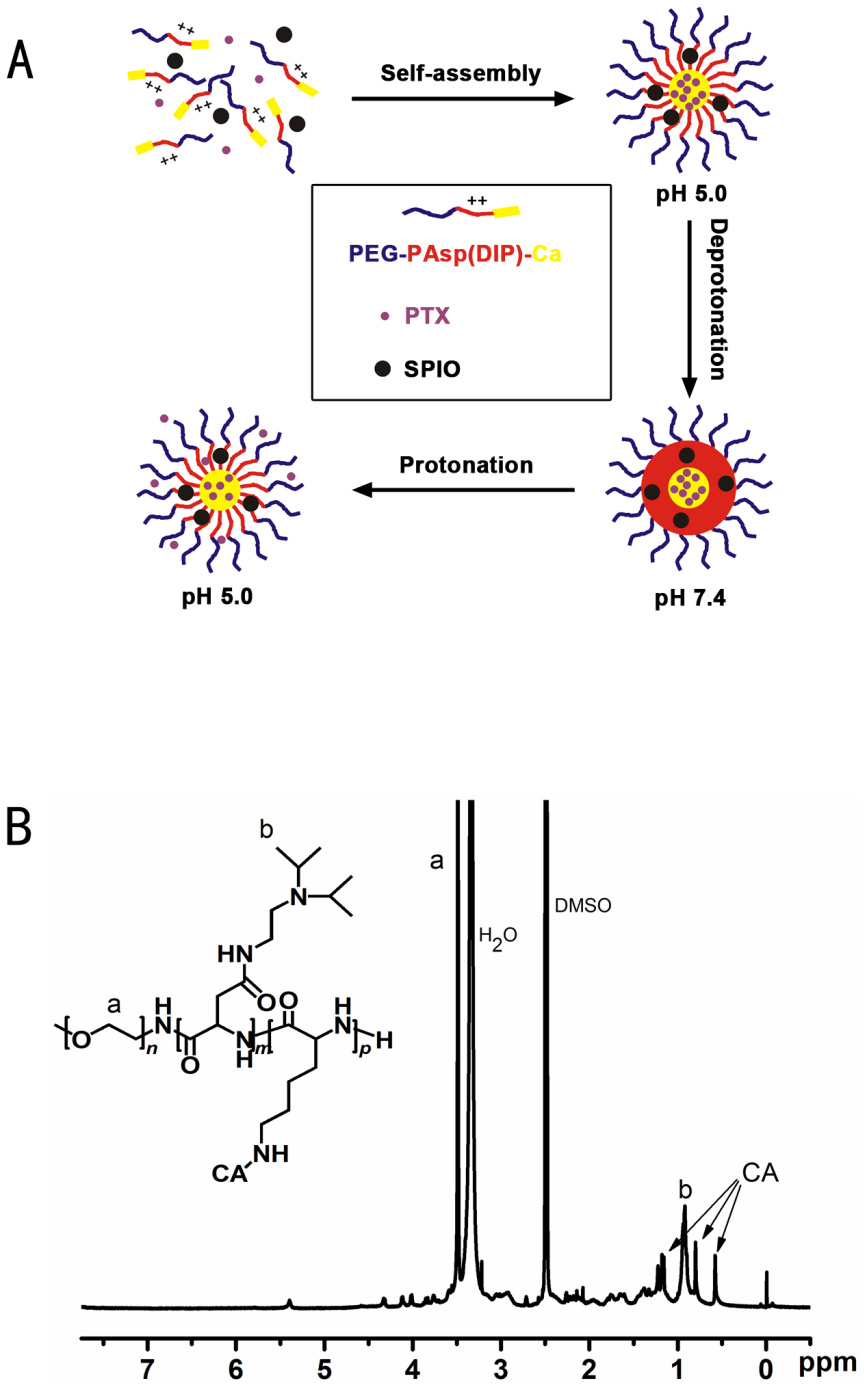
Illustration of PEG-P(Asp-DIP)-P(Lys-Ca):Drawing of pH-tunable drug release (A) and ^1^H-NMR (300 MHz) spectra of PEG-P(Asp-DIP)-P(Lys-Ca) (B).

PEG-P(Asp-DIP)-P(Lys-Ca) and SPIO-PEG-P(Asp-DIP)-P(Lys-Ca) were provided by School of Chemistry and Chemical Engineering (Fig. 1A, B), Sun Yat-Sen University and the micelles was synthesized as previously reported [18]. LoVo cells from a human CRC cell line were purchased from the Institute of Biochemistry and Cell Biology (Chinese Academy of Sciences, Shanghai, China). Taxol was obtained from Bristol-Myers Squibb Co. (Princeton, NJ, USA), Minimum essential medium (MEM) and fetal bovine serum (FBS) were purchased from Invitrogen Corporation (GIBCO, Carlsbad, CA, USA).

### Size and morphology

BI-200 SM dynamic light scattering (DLS) (Brookhaven Instruments Corp., Holtsville, NY, USA) with a 532 nm vertically polarized argon ion laser as the light source, was used to determine the particle size. The measurement was performed at a 90°angle at 25°C and scattered light was collected on a BI-9000AT Digital Autocorrelator (Brookhaven Instruments Corp., Holtsville, NY, USA). The particle size and zeta potential of each sample were measured for five times. Transmission electron microscopy (TEM) images were obtained on a JEOL TEM-2010HR (JEOL Ltd., Japan) operated at 160 kV.

### Intracellular drug delivery and release efficiency measurements

#### Delivery efficiency *in vitro*

LoVo cells were seeded in 6-well plates or petri dishes at a density of 1×10^5^ cells per well/dish and incubated in 2 mL of MEM containing 10% FBS overnight. 50 µL of FDA-SPIO-PEALCa complex solution was added to each well at a final fluorescein diacetate (FDA, the fluorescent substitute of PTX) concentration of 2 µg/mL. The cells were then incubated with the complex solution for 6 h and finally subjected to flow cytometry analysis and confocal laser scanning microscopic (CLSM) observation to determine the intracellular drug delivery efficiency of FDA-SPIO-PEALCa as a hydrophobic drug vector.

#### Flow cytometry analysis

After FDA-SPIO-PEALCa (final FDA concentration of 2 µg/mL) was incubated with the LoVo cells for 6 h, cells were trypsinized and washed with phosphate-buffered saline (PBS), resuspended in 0.5 mL of PBS. FDA content was detected by flow cytometry (FACSCalibur, BD Co, USA) using a 488 nm laser for excitation. The green fluorescence emission of FDA was collected through a 525 nm filter. LoVo cells without transfection were used as a control for background calibration. The flow cytometry data were analyzed using the FlowJo software (Version 7.6, Treestar, Inc., San Carlos, CA).

#### Confocal laser scanning microscopy

The intracellular distribution of hydrophobic green fluorescent FDA was observed on CLSM. Four hours after the incubation with FDA-SPIO-PEALCa, LoVo cells seeded in petri dishes were washed for three times with PBS. To further confirm the intracellular distribution of FDA, the lysosomes and nuclei were stained with red (Lysotracker Red, Molecular Probe, Eugene, OR) and blue (Hoechst 33324, Beyotime Biotech, China) fluorescent dyes, respectively. Cells were observed on a Zeiss LSM 510 META microscope (Carl Zeiss Cl, Ltd, Gottingen, Germany). FDA, Lysotracker Red and Hoechst 33342 were excited at 488 nm, 514 nm and 352 nm respectively, and the corresponding emission wavelengths were 525 nm, 455 nm and 595 nm, respectively.

#### Drug release at different pH values

The *in vitro* drug release behavior of the micelles was determined using a dynamic dialysis method at 37°C. FDA instead of PTX was encapsulated inside the PEALCa micelle. 50 µL of FDA-PEALCa complex solution was adjusted to pH 5.0 with 1 N HCl or pH 7.4 with PBS at a final FDA concentration of 2µg/mL, then the solution was transferred into a dialysis bag (MWCO: 14000). The bag was then placed into buffered solution (40 mL) at the same pH with gentle shaking of 75 rpm (ZHWY-200B, Shanghai Zhicheng, Shanghai, China). At predetermined time intervals (0.5, 1.0, 4, 18 and 24 h, respectively), the solution outside dialysis bag was removed for UV-Vis analysis and replaced with fresh buffer solution. FDA concentration was calculated based on the absorbance intensity at 430 nm. The cumulative amount of released FDA was calculated against release time for assessment of drug release.

### Cytotoxicity and cell apoptosis inducing by PTX-SPIO-PEALCa

#### Biocompatibility assay

Methyl thiazolyl tetrazolium (MTT) assay was performed to evaluate the cytotoxicity of PEALCa and SPIO-PEALCa complexes. Human (CRC) LoVo cells were cultured in MEM supplemented with 10% FBS and incubated at 37 °C in a humidified atmosphere with 5% CO_2_. In MTT assays, the cells were seeded in 96-well plates with the density of 5000 cells/well. Then the cells were incubated in fresh medium with various concentrations of PEALCa or SPIO-PEALCa (0–300 µg/mL) for 24 h. Then the PEALCa- or SPIO-PEALCa-contained MEM was replaced by fresh medium with MTT solution (Sigma, St Louis, MO) at 5 mg/mL and incubated for 4 h; subsequently, 100 µL dimethyl sulfoxide (DMSO) was added to each well to dissolve the formazen after the MTT-contained medium was aspirated. Finally, the absorbance at 494 nm of each well was recorded using the Infinite F200 multimode plate reader (Tecan, Männedorf, Switzerland). Three duplicates were measured for each experimental point.

#### Cell apoptosis and cytotoxicity detections

Hydrophobic chemotherapeutic drug PTX was simultaneously encapsulated with SPIO in the pH-sensitive PEALCa micelle (PTX-SPIO-PEALCa). To determine the LoVo cell-apoptosis inducing ability and chemotherapeutic efficacy of PTX-SPIO-PEALCa, MTT and terminal deoxynucleotidyl transferase-mediated UTP nick end labeling (TUNEL) assays were performed, respectively.

MTT assays were used to compare the cytotoxicity of the LoVo cells between PTX-SPIO-PEALCa micelles and free Taxol. After seeding at a density of 5000 cells per well in 96-well plates and cultured overnight, the LoVo cells were incubated with PTX-SPIO-PEALCa and Taxol of various PTX concentrations (from 0 µg/mL to 5 µg/mL) for 48 h, respectively. The PTX-containing medium was replaced with 100 µL of fresh MEM containing 0.5 µg/mL MTT (Sigma) and subsequently incubated with the LoVo cells for 4 h. Then the medium in each well was aspirated and replaced by 100 µL of DMSO to dissolve the formazen. Finally, after 5 min gentle agitation of the 96-well plates, the absorbance at 494 nm in each well was detected using the Infinite F200 multimode plate reader.

In TUNEL assay, LoVo cells were seeded at a density of 2.5 × 10^4^ per well on coverslips in 6-well plates, normally cultured overnight, and then incubated with PTX-SPIO-PEALCa for 24 h at four PTX concentrations (0, 0.1, 0.5, 1.0 µg/mL). TUNEL assays were carried out using an *in situ* cell death detection kit, peroxidase (POD) (Boehringer Mannheim, Mannheim, Germany) according to the manufacturer's protocol. In brief, cells on coverslips were fixed with freshly prepared 4% paraformaldehyde for 15 minutes (min) at room temperature. After incubated with 0.3% H_2_O_2_ in methanol for 10 min at room temperature, the LoVo cells were subsequently treated with 0.1% Triton X-100 in 0.1% sodium citrate for 2 min on ice for permeation. The permeabilized cells were incubated with 50 µL of terminal deoxynucleotidyl transferase (TdT) reaction mixture in a humidified chamber for 60 min at 37°C. Then the slides were incubated with 50 µL of anti-fluorescein antibody conjugated with POD for 30 min at 37 °C. Finally, diaminobenzidine (DAB) substrate reacting with apoptotic cells to form the brown staining was defined as the positive signal, while the blue negative signal from hematoxylin was applied to identify the non-apoptotic cells. All samples were observed under optical microscope.

For the quantification analysis of TUNEL assays, we randomly selected three low magnification visions in each sample with ImageJ software, and calculated the number of DAB-positive cells and DAB-negative cells within each one of the vision field. The TUNEL positive rate of tumor cells was calculated using the formula:



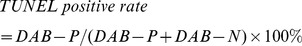
(1)


where DAB-P was the number of DAB-positive cells, and DAB-N was the number of DAB-negative cells, and the average value of each group was calculated.

### *In vivo* studies

This study was carried out in strict accordance with the guidance of the Institutional Animal Care and Use Committee (IACUC) of Sun Yat-Sen University (Permit Number:2013 A-038). The use of chloral hydrate were approved by the IACUC as non-pharmaceutical grade and given special exemption by the institutional IACUC, and all efforts were made to minimize suffering.

#### Human colon carcinoma xenografts model

Human CRC LoVo cells were implanted into the BALB/c nude mice (4–5 weeks) to establish the colorectal carcinoma xenografts models. The mice (*n* = 14) were placed in the stereotactic frame after they were anesthetized with 10% chloral hydrate (400 mg/kg). 1×10^6^ LoVo cells were trypsinized, washed for three times with PBS, resuspended in 50 µL of PBS and subcutaneously injected in the right back side of the mice. The tumor-bearing mice and the tumors were monitored every day. In brief, the length (L) and the width (W) of the tumors were measured with caliper. The tumor volume was calculated as following equation [19]:



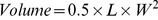
(2)


When the volume of tumor reached about 40 mm^3^ to 60 mm^3^ after 5–7 days, the mice were used for *in vivo* MR imaging and chemotherapy studies.

#### *In vivo* MR imaging

As previously reported, most obvious MRI contrast could be achieved at the injection dose of 4.48 µg Fe/g body weight [20]. Therefore, an injection dose of 4.48 µg Fe/g body weight was used to detect tumors by MRI in our study. Two mice were randomly chosen and anesthetized by intraperitoneal injection of 10% chloral hydrate. T2-weighted MRI was performed prior to injection and repeated at 0.5, 1, 1.5 and 2 h after the injection of 10% SPIO-PEALCA (Group 1) at a dose of 4.48 µg Fe/g body weight and 100 µL 10% blank PEALCA (Group 2) intravenously through the tail vein, respectively, on a 3.0 T MRI scanner (Magnetom Avanto; Siemens Healthcare Sector, Erlangen, Germany) with a 5-cm linearly polarized birdcage radio frequency mouse coil. The T2-weighted images were acquired using a fast spin-echo sequence with these parameters: repetition time 2000 ms, echo time 80 ms, FOV 3×3 cm, slice thickness 1.0 mm and flip angle 90°.

#### Anticancer efficacy studies

In tumor therapeutic experiments, the remaining LoVo tumor-bearing mice were randomly divided into three groups (n = 4 for each group) as Group 1, 2, and 3. In group 1, which was defined as the experimental group, the mice were injected with PTX-SPIO-PEALCa; In Group 2, defined as the experimental control group, the mice were injected with the commercial cremophor-based PTX (Taxol); and in Group 3, defined as the negative control group, the mice was injected with PBS. To demonstrate the tumor targeted PTX delivery efficiency of the pH-sensitive PEALCa micelles compared with commercial PTX preparations, a lower final PTX plasma concentration was used for *in vivo* chemotherapeutic studies. PTX-loaded micelles PTX-SPIO-PEALCa, Taxol and PBS were intravenously injected via tail veins after dilution in normal saline to 100 µL. PTX concentration in both PTX-SPIO-PEALCA group and Taxol group were 1 mg/kg body weight. As the first drug intervention day was defined as day 0, the drug was administrated on day 0, day 2, and day 4. Tumor volumes, living conditions and survival situations in all groups were recorded every two days.

In day 35, mice in all groups were sacrificed and then tumors were collected, fixed in 10% formalin for 24 h and embedded in paraffin. Haematoxylin/eosin (H&E) staining of tumor tissue sections (3 µm) was performed for the observations of cancer tissue morphology in different groups. To better elucidate the anticancer effects and apoptosis inducing ability of the PTX-loaded micelles PTX-SPIO-PEALCa, *in situ* TUNEL assays were carried out on the excised tumor tissue. The TUNEL assays were performed according to the manufacturer's protocol using a FragELTM DNA Fragmentation Detection kid (EMD chemicals Inc, Darmstadt, Germany). Tumor tissue sections were deparaffinized, and permeated using proteinase K (20 µg/mL). After the H_2_O_2_ aqueous solution (3%) was added to inactivate endogenous peroxidase, the slides were treated with TdT Enzyme at 37 °C for 90 min. The slides were finally incubated with streptavidin-horseradish peroxidase conjugate, during which the DAB reacted with the apoptotic cells to form the brown signal, while non-apoptotic cells were counterstained as blue-green to be distinguished from the apoptotic signal.

### Statistical analysis

The tumor size of three groups were presented as mean ± standard deviation and analyzed by the Student's *t*-test (SPSS software, Version 13.0, SPSS Inc.). A two sided *P* value less than 0.05 was considered statistically significant.

## Results

### Micelle size

**Figure 2:**
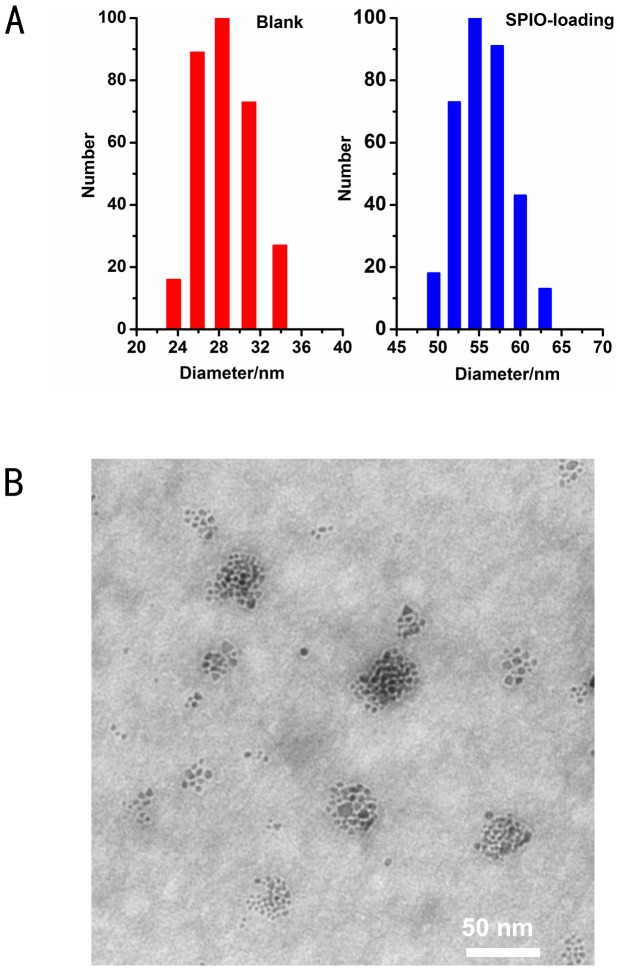
Micelle size measure. Size distribution of PEALCa micelles before and after loading of SPIO(A). Transmission electron microscopy (TEM) image of SPIO-loaded micelle (B). The scale bars represent about 50 nm.

The micelle size was about 29 nm before SPIO loading and 54 nm after SPIO loading (Fig. 2 A). TEM images showed that the SPIO-loaded micelles were spherical with an average size of 50 nm (Fig. 2 B).

### Intracellular drug delivery and release efficiency measurements

#### Delivery efficiency *in vitro*

**Figure 3:**
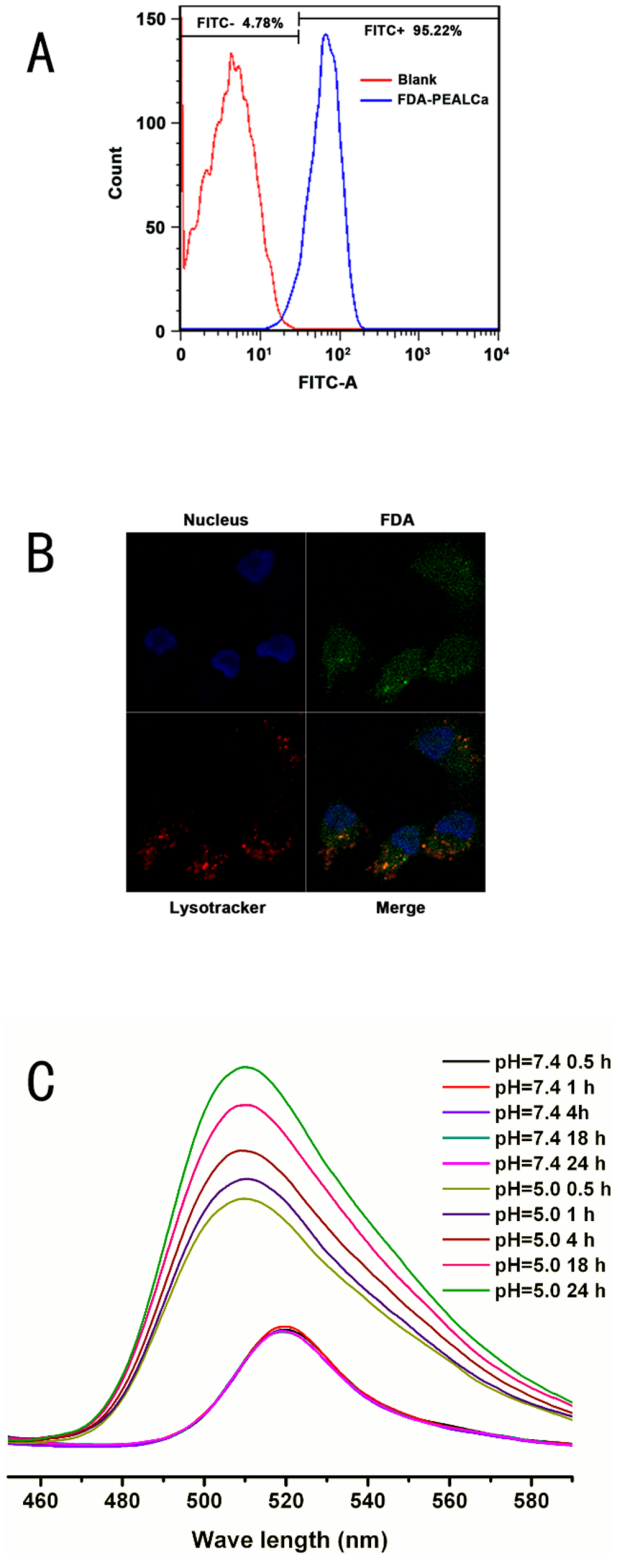
Intracellular drug delivery and release efficiency measurements. Quantitative analysis of FDA-positive LoVo cells by flow cytometry after incubation with FDA-SPIO-PEALCa (A). Confocal laser scanning microscopy (CLSM) images (1000 ×) of LoVo cells after treated with FDA-SPIO-PEALCa (B). The lysosomes and nuclei were stained by red and blue fluorescent probe, respectively. *In vitro* FDA released from PEALCa micelle at 37 °C in HCl (pH 5.0) and PBS (pH 7.4) (C).

The flow cytometry analysis of LoVo cells was carried out 6 h after the incubation with FDA-SPIO-PEALCa. The green fluorescent positive ratio of LoVo cells was 95.2±3.7%, and that of the negative untreated cells was 4.78±4.6% (Fig. 3A).

The uptake and intracellular distribution of FDA was detected by CLSM observation. To better illustrate the distribution of FDA, the lysosomes and nuclei were stained with red and blue fluorescence as the markers for intracellular localization. Figure 3B showed that after 4 h of incubation with FDA-SPIO-PEALCa, the green fluorescence of FDA was visible both in cytoplasm and nucleus. Especially in red fluorescent lysosomes, higher amount of FDA fluorescence was detected.

#### Drug release *in vitro*

Drug release test were carried out at pH 5.0 and 7.4, respectively. The result showed that the maximum fluorescence intensity of pH 5.0 at each time interval was higher than that of pH 7.4. Furthermore, fluorescence intensity of pH 7.4 group had no obvious change throughout the experimental time of 24 h (Fig. 3C), implying that micelle structure were stable in neutral condition, with only a small amount of FDA released. In contrast, fluorescence intensity of pH 5.0 significantly increased against time, revealing that FDA release was both time- and pH- dependent.

### Cytotoxicity and cell apoptosis inducing by PTX-SPIO-PEALCa

#### Biocompatibility assay

**Figure 4:**
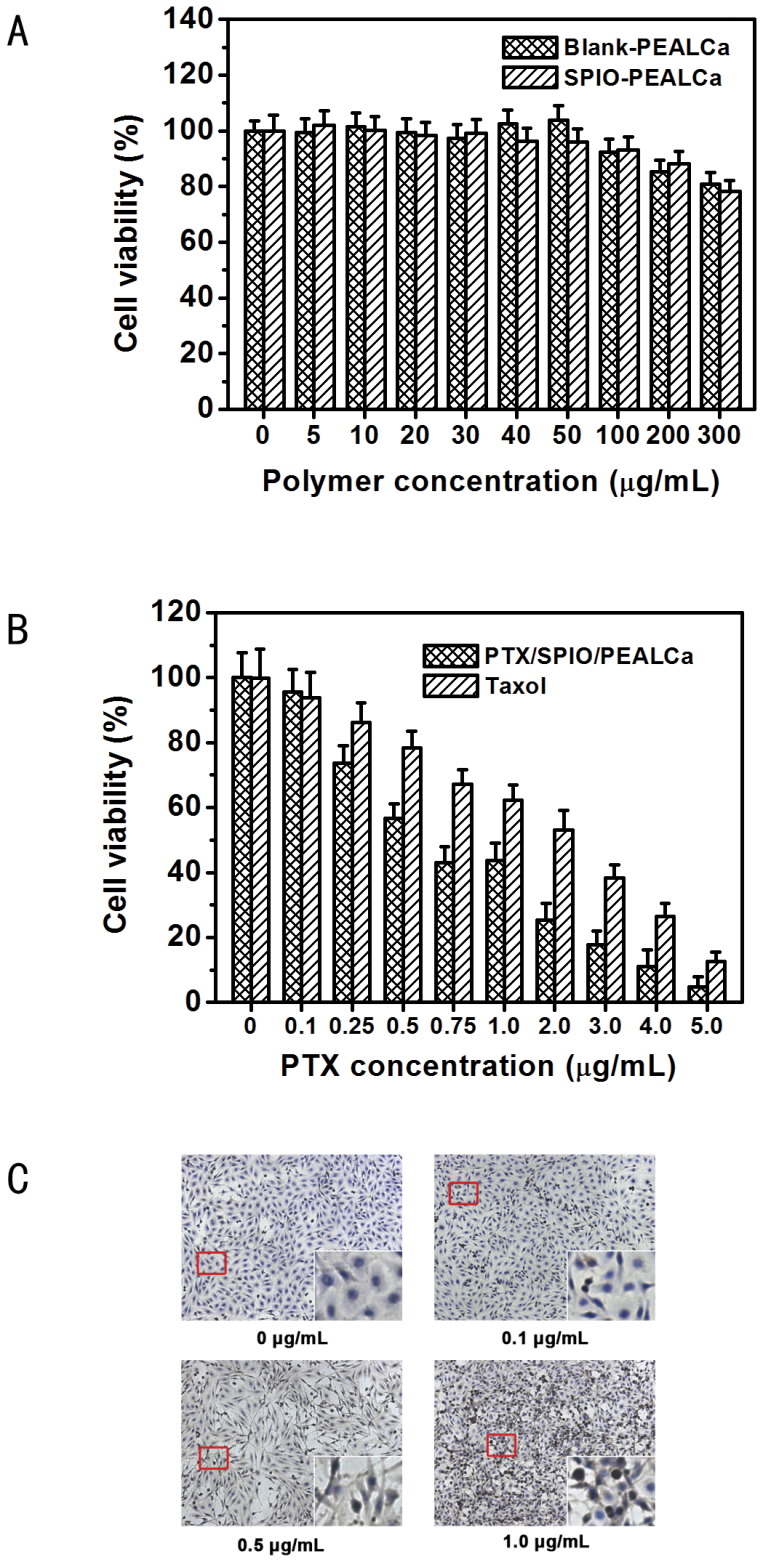
Cytotoxicity and cell apoptosis evaluation. *In vitro* cytotoxicity of various concentrations of PEALCa and SPIO-PEALCa in LoVo cells detected by MTT assay (A). MTT assay of the cytotoxicity of PTX-SPIO-PEALCa and free Taxol with different PTX concentrations (B). LoVo cell apoptosis detected by *TUNEL* assay after PTX-SPIO-PEALCa incubation with different PTX concentrations (50×) (C). Magnified image of the red rectangular represents the representative normal or apoptotic cell.

The cytotoxicity of nano-complexes was evaluated *via* MTT assay. As shown in Figure 4A, the viability of tumor cells incubated in blank micelles decreased gradually. Even at high polymer concentration (300 µg/mL), the viability of LoVo cells remained at a high level (81.0±4.0%). Meanwhile, the pH-sensitive micelles loaded with SPIO also revealed low cytotoxity. All these results illustrated that PEALCa was safe for bio-applications and the presence of SPIO did not increase the cytotoxicity of the copolymers.

#### Cytotoxicity and apoptosis inducing detections

MTT assay was conducted to further compare the cytotoxicity of the pH-sensitive PTX-SPIO-PEALCa micelles and free Taxol (Fig. 4B). The results revealed that the cell viability decreased along with the increase of PTX concentration. Moreover, under the same PTX concentration, the cell viability of PTX-SPIO-PEALCa micelles was lower than that of the free Taxol. The IC50 value of PTX-SPIO-PEALCa and free Taxol were 1.27 µg/mL and 2.40 µg/mL, respectively. The result of TUNEL assay also confirmed similar phenomenon (Fig. 4C). Quantitative analysis of TUNEL assays revealed that the apoptosis rate induced by PTX-loaded nanoparticles were 0.7±0.5%, 7.4±0.8%, 19.7±1.1% and 48.8±2.3% for 0 µg/mL group, 0.1µg/mL group, 0.5 µg/mL group and 1.0 µ/mL group, respectively.

### *In vivo* studies

#### MR scanning

**Figure 5:**
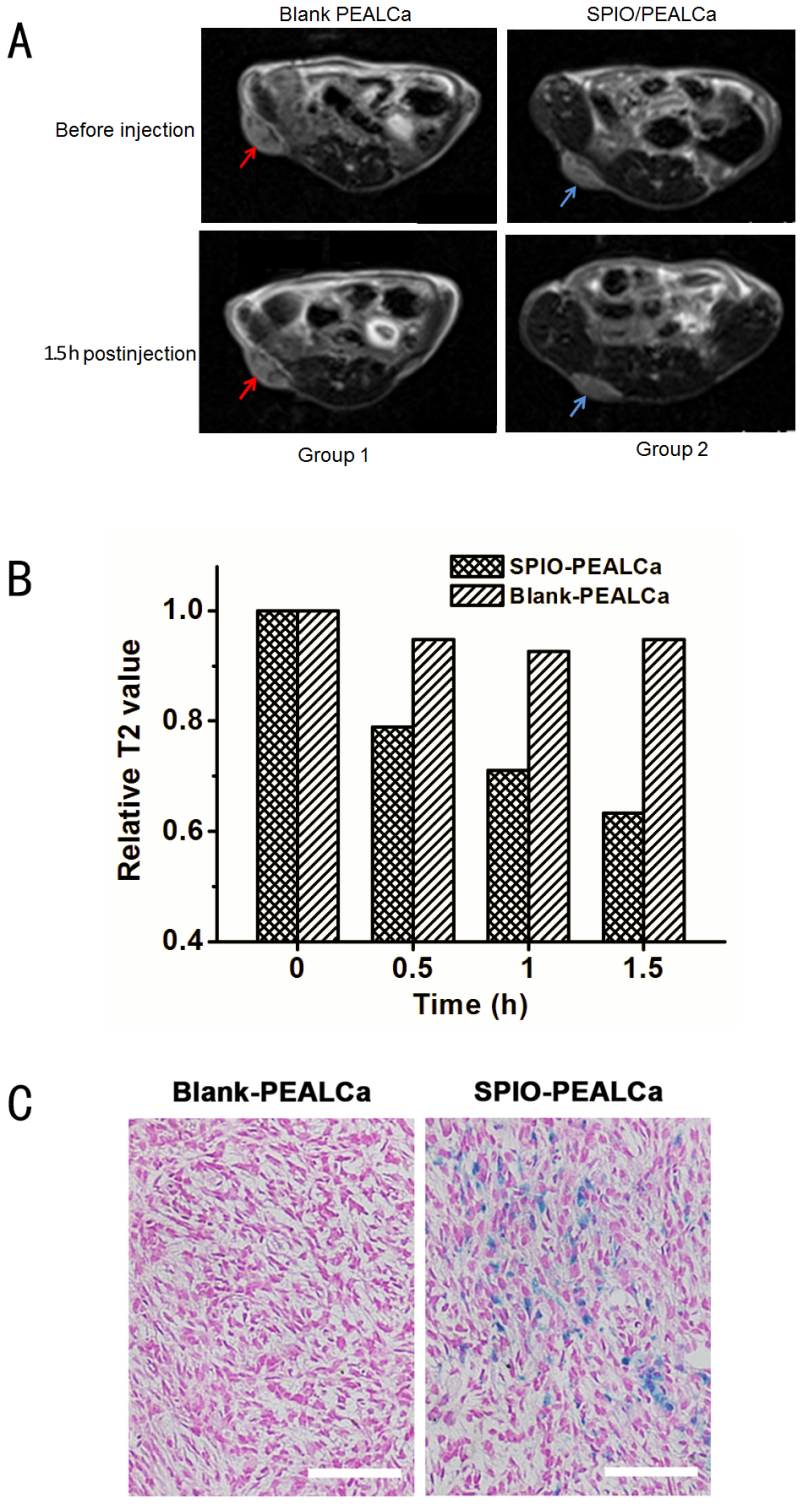
Tumor targeting evaluation *in vivo*. Group 1: mice with LoVo tumors prior and after injection of blank PEALCa; Group 2: mice with LoVo tumors prior and after injection of SPIO-PEALCa (A). The relative T2 value of both groups prior and after injection(B). Prussian blue staining images of the tumor histological sections treated with PTX-PEALCa and PTX-SPIO-PEALCa (200 ×) (C).



To validate the tumor targeting ability of SPIO-PEALCa, two mice bearing LoVo colorectal tumors were randomly chosen in the study. Both mice were injected with blank (group 1) or targeted (group 2) polymers. Prior to injection, both tumors marked with red and blue arrows (Fig. 5A) exhibited hyperintense areas on T2-weighted MRI images. 0.5-1.5 h after injection of blank PEALCa, the signal intensity of tumors showed no obvious drop. However, the T2 signals of the tumors injected with SPIO-PEALCa dropped obviously after 1.5 h of injection. We calculated the Relative T2 value using the formula:

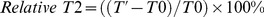
(3)


where T′ was the T2 value of the tumor at different time after injection, and T0 was the T2 value prior to injection. (Fig. 5B).

#### Prussian blue staining

The two mice above were then sacrificed for Prussian blue staining of tumor paraffin sections after the SPIO-PEALCa intravenous injection for explanation of the MR signal reduction in tumor tissue. Prussian blue staining in the sections of blank PEALCa group was rare. On the contrary, the sections from SPIO-PEALCa group showed strong Prussian blue staining in large area of the visual field, indicating that high amount of SPIO was delivered into the tumor tissue by the pH-sensitive micellar nanoparticles (Fig. 5C). The results were consistent with the above in vivo MRI image findings.

#### Anticancer efficacy *in vivo*

**Figure 6:**
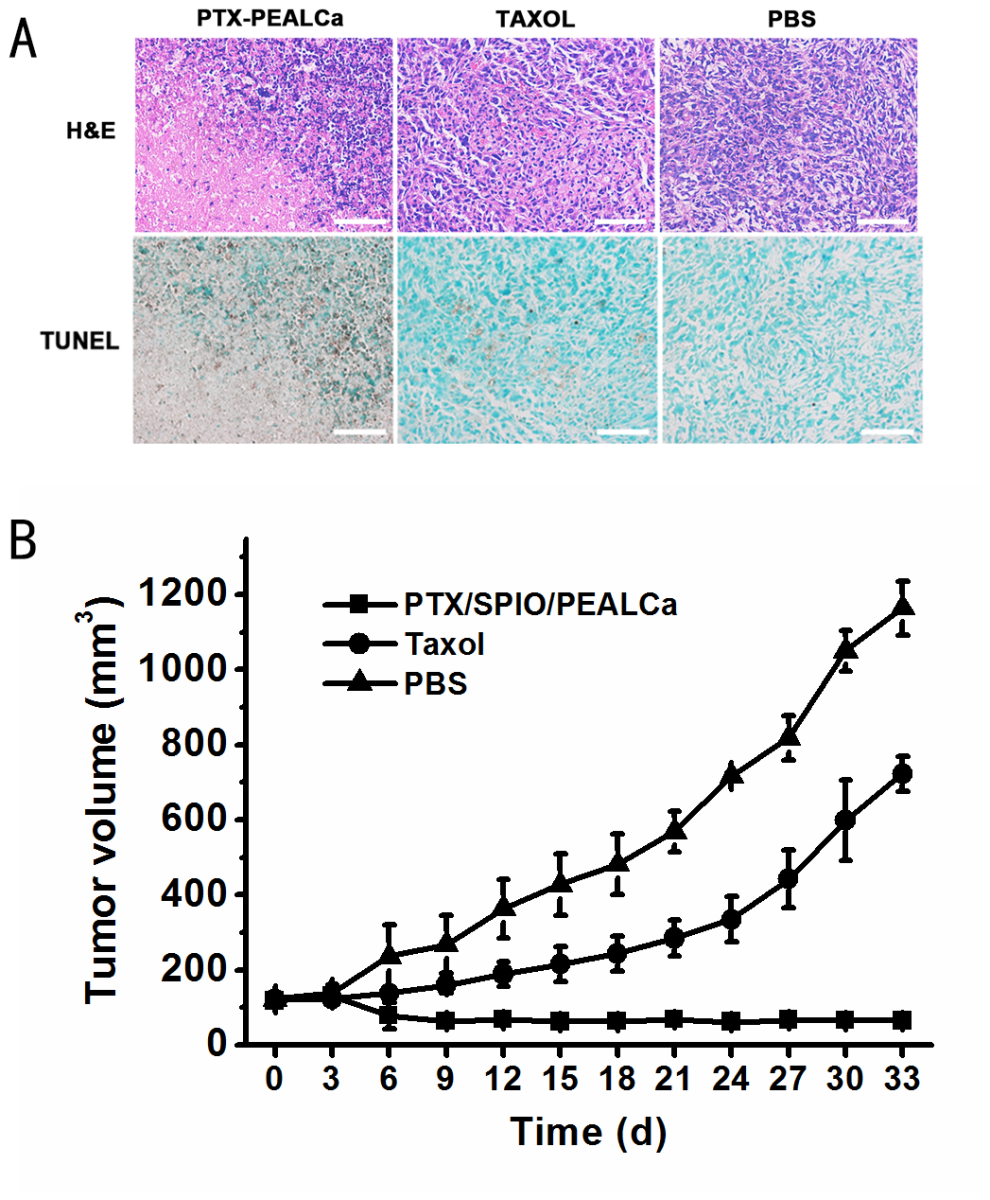
Anticancer efficacy evaluation *in vivo*. Representative haematoxylin and eosin (H&E) and terminal deoxynucleotidyl transferase-mediated UTP nick end labeling (TUNEL) staining images (200 ×) of tumor sections from the mice of different groups(A). Tumor growth curves of the LoVo tumor bearing mice treated with PTX/SPIO/PEALCa, Taxol or PBS (B).

To further elucidate the anticancer efficacy of PTX-loaded micelle *in vivo*, the remaining 12 mice were used for tumor apoptosis inducing study. After 35 days of the first treatment, the tumor tissues of 3 groups were paraffin sectioned and stained with H&E to evaluate the histological changes of the tumors. As shown in Figure 6A, more hypercellular and nuclear polymorphism were observed in the H&E stained sections of the PBS negative control group. On the contrary, the morphology of PTX-SPIO-PEALCa group demonstrated more hypocellular, nuclear shrinkage, and the highest level of tumor cell apoptosis and necrosis. The Taxol control group showed medium level of cell density and nuclear atypia. Furthermore, TUNEL assay results confirmed that the TUNEL-positive cells in PTX-SPIO-PEALCa group were significantly higher than the Taxol group, indicating that the tumor apoptosis inducing efficacy of the pH-sensitive micelles was much higher than that of commercial chemotherapeutic agent.

The tumor growth curves also illustrated the therapeutic effects of PTX-loaded micelles in general (Fig. 6B). Tumor volume of the PTX-SPIO-PEALCa group remained in a low level during the whole experimental process, which meant that the tumor growth of the PTX-SPIO-PEALCa group was almost negligible. The averaged tumor volume of the PTX-SPIO-PEALCa group was 65.0±8.4 mm^3^ at day 30, which was significantly smaller than the Taxol group of 598.7±77.4 mm^3^ and the PBS group of 1050.7±54.4 mm^3^ (*t* = 5.34, *P*<0.001).

## Discussion

PTX is an effective chemotherapeutic agent by stabilizing microtubules and mitotic arrest. It has shown broad-spectrum activity in solid tumors, including colorectal cancer [21], [22]. Despite the clinical advantages of PTX, it has a low therapeutic index because of hydrophobic property and serious side effects. As a result, the main challenge in PTX chemotherapy is the development of safe and highly efficient delivery systems that can encapsulate and transfer PTX into cancerous cells with lower systemic toxicity and better anti-tumor efficiency. It is well known that the tumor vasculature has poor architecture due to abnormal basement membrane and fissures between the endothelial cells accompanied with a poor lymphatic drainage system. This state of leaky vasculature causes that particles with size ranging 10 to 100 nm can preferentially accumulate in the tumors and have longer circulation time, unlike the free drugs, which are of much smaller size but may easily deposit in the normal tissue and be cleared by the kidney [23], [24]. Notably, TEM observation in our study showed that the PTX-loaded micelles possessed a uniform small size of around 54 nm, which is highly desirable for longer blood circulation time and easier cellular uptake by cancer cells [25], [26].

In this study, the PEG-P(Asp-DIP)-P(Lys-Ca) (PEALCa) triblock copolymer was used as an essential component of drug delivery vehicle, and its characteristics under MRI imaging as well as anti-tumor efficay were investigated. Drug release profiles revealed that rate of FDA release from PEALCa micelles was both time- and pH- dependent. In a neutral environment (pH = 7.4), micelle structure was stable and FDA was steadily released from the micelles. Nonetheless, a rapid and much more intensive release of FDA was turned on immediately at acidic environment (pH = 5.0). When the micelle enters cells *via* endocytosis, the experienced pH drops from neutral to as low as 5.5-6.0 in endosomes and 4.5–5.0 in lysosomes and triggers rapid drug release. In micelles, PEG forms an outer corona stabilizing the micelles to avoid uptake of reticuloendothelial system [27], [28]. P(Asp-DIP) is a pH-sensitive interlayer that undergoes hydrophobic-hydrophilic transition in the acidic lysosomes of cells [29]. Cholic acid (Ca) forms the micelle core to encapsulate hydrophobic drugs and also contribute to rapid drug release [18], [30]. Our results suggested that PEALCa micelle can be used as a nanocarrier of PTX, which can delay drug release and absorption in normal tissue and enhance transportation of the drug to human CRC LoVo cells and improve the anti-tumor efficiency.

MTT assay was used to determine the cytotoxicity of the vectors. With increased polymer concentration, the viability of LoVo cells in the blank and SPIO-loaded micelles decreased gradually. Acceptable cytotoxicity was achieved when polymer concentration remains below 300 µg/mL. It is well known that SPIO is one of the most effective MRI contrast agents and possesses a high biocompatibility and low toxicity [16], [17]. The pH-sensitive micelles encapsulated with SPIO also exhibited low cytotoxity. The results revealed that the copolymers had little cytotoxicity to LoVo cells. Moreover, under the same PTX concentration, the cell viability treated with PTX-SPIO-PEALCa micelles was lower than that of free Taxol, which revealed the cytotoxicity of PTX-SPIO-PEALCa micelles was higher than free Taxol. The TUNEL assay clearly demonstrated significantly high levels of apoptosis in LoVo cells as the concentration of targeting polymers increased.

Quantitative flow cytometry results showed that the intracellular delivery of hydrophobic fluorescent dye FDA was highly efficient. The uptake and intracellular distribution of FDA was also detected by CLSM observation, as FDA was visible both in cytoplasm and nucleus, especially in red fluorescent lysosomes, which was consistent with the flow cytometry analysis findings. This assay verified that PEG-P(Asp-DIP)-CA lead to the efficient release of FDA in the LoVo cells.

Encouragingly, the *in vivo* tumor targeting ability of PTX-SPIO-PEALCa was validated by MRI technology. After SPIO-PEALCA or blank PEALCa micelle were administrated through tail intravenous injection in the mice bearing LoVo tumor (right thigh), the tumor signal intensity and relative T2 value of the mouse injected with SPIO-PEALCa had dropped significantly as revealed by MRI scans. However, the mouse injected with blank PEALCa failed to demonstrate such drop in signal intensity or relative T2 value at tumor site. Consistent results were observed by Prussian blue staining, indicating that the hypointensity of the tumor site was due to the intracellular SPIO delivered by PEALCa. Furthermore, the *in vivo* studies revealed a significant delay in tumor growth with PTX-SPIO-PEALCa micelle therapy in mice. All of the above results illustrated that the PEALCa micelle delivered PTX and SPIO into LoVo cells effectively and has assisted an efficient release of PTX, thus promoted the apoptosis of LoVo cells.

In recent years, several representative pH-sensitive polymeric micelles have been reported as potential cancer diagnostics and therapies. A pH-sensitive biodegradable poly (β-amino ester) (PAE) was developed by Langer and coworkers [31]. Fe_3_O_4_-loaded PEG-b-PAE micelles are stable at 37°C and pH 7.4 but rapidly disassembles at pH below 7.0 [32],resulting in drug release in the extracellular space of tumor (pH =  6.5–7.2) [33] and leading to a reduction of the intracellular release and consequently decreased the therapeutic efficacy of anti-tumor drugs. Poly (L-histidine) (PHis) is another pH-sensitive polymer suitable for cancer therapy with excellent biocompatibility [34]. However, PH is too sensitive to environmental pH, which could affect the stability of the core of the drug carrier. Other potential pH-sensitive polymeric micelles for cancer diagnosis and therapy have been also developed based on pH-sensitive groups, including poly (malic acid) [35], chitosan [36], histidine side groups [37] and so on. In summary, a novel pH-sensitive nanoparticulate vector with PTX delivery and MRI functions was fabricated by entrapping PTX and SPIO into PEALCa micelle. The pH-triggered drug release was found to efficiently amplify the intracellular drug concentration which determined the anticancer outcome. The drug-delivery effect *in vivo* was visualized by MRI technology. The results of this study revealed the great potential of PTX-SPIO-PEALCa micelle as a multifunctional nanomedicine for tumor therapy.

The main limitation for the study is that we only use one CRC cell line (LoVo) to evaluate the efficiency of PEALCa in controlled and efficient drug delivery. Our future study will focus on monitoring the drug transferring of PEALCa and anti-cancer therapeutic efficiency of PTX-PEALCa on larger sample size of animals,other CRC cell lines and other tumors, in order to further validate our findings.
